# A bifunctional nonenzymatic flexible glucose microsensor based on CoFe-Layered double hydroxide†

**DOI:** 10.1039/c8na00231b

**Published:** 2019-01-10

**Authors:** Junya Cui, Zhenhua Li, Ke Liu, Jianming Li, Mingfei Shao

**Affiliations:** State Key Laboratory of Chemical Resource Engineering, Beijing University of Chemical Technology Beijing 100029 China shaomf@mail.buct.edu.cn +86-10-64425385 +86-10-64412131; Petroleum Geology Research and Laboratory Center, Research Institute of Petroleum Exploration & Development (RIPED), PetroChina Beijing 100083 China

## Abstract

A bifunctional flexible glucose microsensor has been successfully fabricated by directly growing a layered double hydroxide nanosheet array (LDH-NSA) on Ni wire. The as-obtained CoFe-LDH-NSA exhibits promising performances in electrochemical and colorimetric detection of glucose with high sensitivity and selectivity. This work demonstrates an effective strategy to fabricate multi-functional glucose nonenzyme sensors.

## Abstract 2

A bifunctional glucose microsensor has been successfully fabricated by electrosynthesis of a CoFe-LDH nanosheet array on the Ni wire.

## Graphical abstract

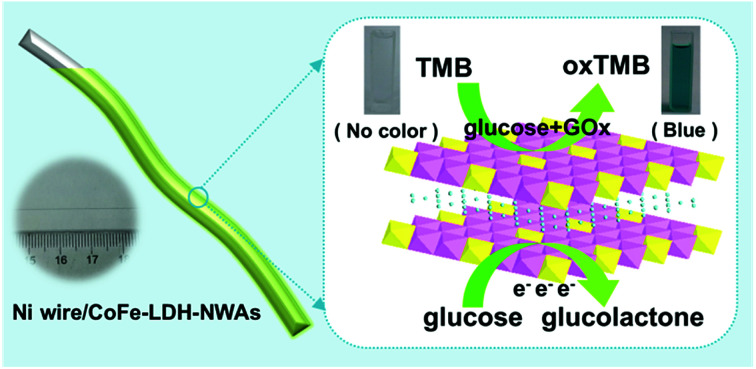

The development of fast responsive, reliable, sensitive and selective strategies for glucose determination is of considerable urgency in biotechnology, diagnosis and food industries.^1–3^ Numerous analytical techniques such as fluorescence,^4–6^ electrochemiluminescence^7–9^ and electrochemistry^10–12^ have been developed to realize this goal. Among them, electrochemical glucose sensors and colorimetric sensors have attracted increasing interest. However, for a given sensor based on mono-electrochemical detection, the fast response and readable signal to the naked eyes are hard to be simultaneously achieved, which limits its applications in qualitative self-help tests. In contrast, colorimetric sensing by transforming the detection events into colour changes provides a facile method independent of instruments,^13,14^ but usually suffers from low sensitivity and reliability. A bifunctional glucose sensor combines the advantages of the electrochemical and colorimetric methods and sheds light on a new generation of high performance glucose sensors.^15^ However, it is still challenging to rationally design and fabricate a bifunctional glucose sensor with enhanced sensitivity, selectivity and reliability.

Layered double hydroxides (LDHs) are layered anionic clays that are assembled by positively charged hydroxide layers ([M^II^_1−*x*_M^III^_*x*_(OH)_2_]^*z*+^; M^II^ and M^III^ are divalent and trivalent metals) and charge compensating interlayer anions.^16–18^ Owing to their specific layered structure and tunable compositions with atomically dispersed transition metals (*e.g.*, Co, Ni, Fe, Cu, Mn, Zn, and Ti) in the host layers, LDHs have been used as amperometric or potentiometric sensors with advantages of nontoxicity, high activity and stability. For example, conductive substrate supported NiAl-, CuAl-, NiFe-, CoFe-, CoNi-LDH have been reported as highly sensitive electrochemical glucose sensors.^19–24^ Besides, some studies confirm that LDH nanopowders (such as CoFe- and NiFe-LDH) can be applied for colorimetric detection of glucose.^25,26^ It will be great significance for simultaneous implementation of colorimetric and electrochemical sensors based on one LDH-based electrode but has been rarely reported. Moreover, it is promising to further construct a microsensor for glucose detection benefiting from the easy fabrication of LDHs on macro-/micro-substrates.^18^

Herein, we design a flexible bifunctional glucose sensor by using a 3D hierarchical CoFe-LDH nanosheet array (CoFe-LDH-NSA) as the catalyst and Ni wire as the microsubstrate, which realizes high-efficiency electrochemical and colorimetric glucose detection (Scheme 1). Remarkably, the Ni wire supported LDH-NSAs (Ni wire/CoFe-LDH-NSA) exhibit high activity and long-term durability for both electrochemical and colorimetric glucose detection with a linear range from 10 to 1000 μM and 1 to 20 μM, respectively. The good performances of the Ni wire supported LDH structure are due to the following reasons: firstly, this integrated electrode facilitates the separation and reuse of active materials in comparison to the traditional powdered samples, especially used in colorimetric glucose sensors; secondly, the uniformly and vertically growing LDH nanosheets on the Ni wire promote the exposure of active sites and provide convenient ion/electron transport channels for electrochemical detection of glucose; thirdly, the Ni wire as the electrode shows great promise for its application in integration and processing of microsensing devices.

**Scheme 1:**
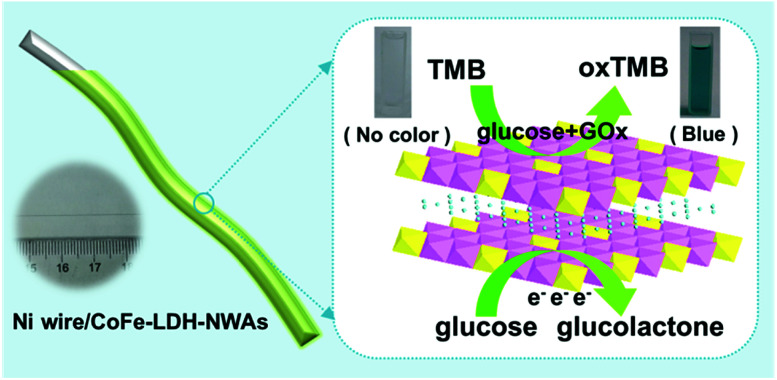
Schematic illustration of the Ni wire supported CoFe-LDH-NSA and the electrochemical and colorimetric glucose detection.

The CoFe-LDH-NSA is directly synthesized on the Ni wire *via* a facile electrosynthesis method at room temperature by using an aqueous solution containing Co(NO_3_)_2_·6H_2_O and FeSO_4_·7H_2_O as electrolytes.^27^ The scanning electron microscopy (SEM) images in Fig. 1A and B show that high density CoFe-LDH nanosheets with a lateral length of 250−300 nm and a thickness of ∼8 nm are uniformly and perpendicularly grown on the surface of the Ni wire after an electrosynthesis time of 100 s. These vertical nanosheets form a three dimensional interconnected network with an open and porous structure. The energy dispersive X-ray spectroscopy (EDS) demonstrates the existence of Co, Fe, O, and C which are homogeneously distributed on the surface of the Ni wire with an elemental content of 7.64%, 7.30%, 70.84%, and 14.22%, respectively (Fig. 1C and S1†). The mass loading of CoFe-LDH on the Ni wire is 0.45 mg cm^−2^. The X-ray diffraction (XRD) pattern of the Ni wire/CoFe-LDH-NSA shows typical diffraction peaks at 11.6°, 23.5° and 34.8°, corresponding to the (003), (006) and (009) reflections of the LDH material (Fig. 1D). No other crystalline phase was detected, indicating the high purity of the LDH-NSA. Moreover, the valence of Co and Fe in the CoFe-LDH-NSA was determined by high-resolution XPS spectra. The high resolution Co 2p_3/2_ XPS spectrum displays a typical peak at 781.6 eV, indicating the existence of Co^2+^ (Fig. S2A†); the binding energies of Fe 2p appear at 712.4 eV (Fe 2p_3/2_) and 725.3 eV (Fe 2p_1/2_), corresponding to the Fe^3+^ oxidation state (Fig. S2B†).

**Fig. 1:**
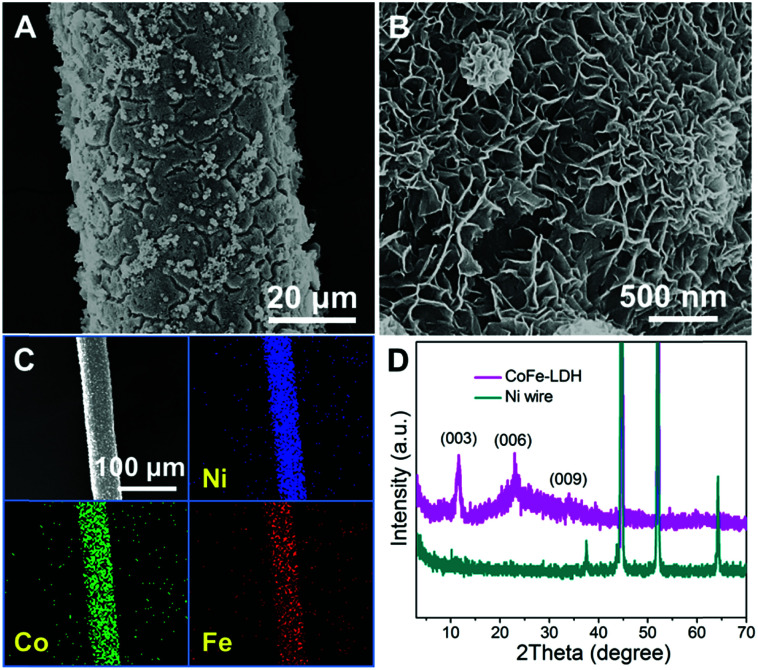
SEM images of the Ni wire/CoFe-LDH-NSA with (A) low and (B) high magnification, respectively. (C) EDS mapping images of the Ni wire/CoFe-LDH-NSA. (D) XRD patterns of the Ni wire and Ni wire/CoFe-LDH-NSA.

The as-fabricated Ni wire/CoFe-LDH-NSA is directly used as a microelectrode to detect glucose. The electrochemical properties are evaluated in 0.1 M KOH solution using a standard three electrode system. As shown in Fig. 2A and S3,† the cyclic voltammogram (CV) curves show a significantly increased anodic current at +0.4 V with the addition of glucose from 0 mM to 3 mM, highlighting the remarkable electrocatalytic activity of the CoFe-LDH-NSA. Fig. 2B is the amperometric response of the Ni wire/CoFe-LDH-NSA for the successive addition of glucose in 0.1 M KOH. The amperometric signals respond quickly to the change in both high and low glucose concentrations (100 μM and 10 μM, respectively), and the calibration curve of current *versus* concentration is plotted in Fig. 2C. A wide linear range is obtained from 10 μM to 1000 μM (*R* = 0.999), with an ultrahigh sensitivity of 1.063 μA cm^−2^ μM^−1^ and a detection limit of 0.27 μM (based on 3 SD/N). These analytical parameters are satisfactory and the linear response range is suitable for the determination of the glucose level in the blood or urine sample.^28^ To confirm the active site in the CoFe-LDH-NSA, we tested the glucose sensing performance of the pure FeOOH-NSA and Co(OH)_2_. As shown in Fig. S4,† the FeOOH electrode shows very weak sensing activation in the presence of 5 mM glucose in comparison with CoFe-LDH, indicating the Co species plays a more important role in the ECA-CoFe-LDH material. However, it has been reported that the existence of the Fe species in LDH would modify the coordination electron structure of LDH host layers and thus enhance the activity and electron transfer.^29,30^ This is also confirmed in this work, as CoFe-LDH possesses a more pronounced current response than pure Co(OH)_2_. Besides, it should be noted that the Ni wire gives negligible contribution to the sensing performance (Fig. S4†). The electrocatalytic glucose oxidation mechanism of CoFe-LDH is tentatively expressed as follows:^19^

1 LDH-Co(ii) + OH^−^ → LDH(OH^−^)Co(iii) + e^−^

2 LDH(OH^−^)Co(iii) + glucose → LDH-Co(ii) + glucolacton

**Fig. 2:**
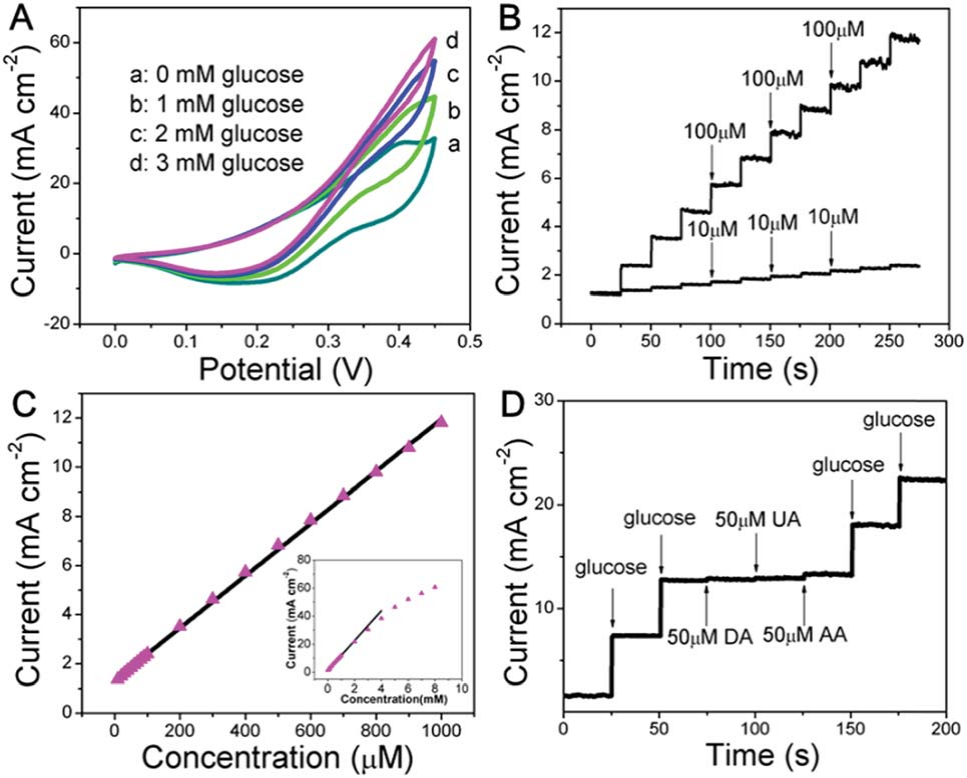
(A) CV curves of the Ni wire/CoFe-LDH-NSA in 0.1 M KOH solution at a scan rate of 5 mV s^−1^ with glucose concentrations from 0 mM to 3 mM. (B) Current–time curves measured at +0.4 V with successive addition of glucose in 0.1 M KOH and (C) the corresponding calibration curve for the electrode. (D) Amperometric response of glucose (0.5 mM) in the absence and presence of 50 μM UA, AA and DA in 0.1 M KOH solution at +0.4 V.

Anti-interference properties are an important criterion for a sensor. Fig. 2D shows the chronoamperometric response of the CoFe-LDH-NSA in the presence of uric acid (UA; 50 μM), ascorbic acid (AA; 50 μM) and dopamine (DA; 50 μM) in 0.1 M KOH solution containing glucose (0.5 mM). It is observed that the current response of glucose does not impose any obvious influence after adding these interferents. Moreover, the Ni wire/CoFe-LDH-NSA electrode also exhibits good stability of up to 10 times of the test using one electrode with a relative standard deviation (RSD) less than 2.2% (Fig. S5A and B†). The catalytic selectivity and stability for the oxidation of glucose is superior to most of the LDH-based materials in the literature (Table S1†). In addition, this CoFe-LDH-NSA is also suitable to detect glucose in a weak alkaline or neutral buffer solution which is closer to the biological environment (Fig. S6†). This Ni wire/CoFe-LDH-NSA can also be used to detect other common saccharides, such as fructose, galactose, lactose, sucrose, and maltose, demonstrating its wide-spread application in carbohydrate sensing on different occasions.

To construct a multi-functional glucose sensor, the colorimetric activity of this hierarchical LDH-NSA for glucose was further studied. Glucose-oxidase (GOx) is used to catalyze the oxidation of glucose with oxygen to form H_2_O_2_.^31^ 3,3′,5,5′-Tetramethylbenzidine (TMB) is employed as the chromogenic reagent, which can be catalyzed by CoFe-LDH from colorless to sky blue (maximum absorbance 652 nm) in the presence of H_2_O_2_ (Fig. S7†). As shown in Fig. 3A (line b), the photograph of the mixed solution containing TMB, GOx and glucose gives no color presentation in the absence of the LDH-NSA. After immersing the Ni wire/CoFe-LDH-NSA in the above solution, a blue color is observed, with a strong absorption band at 652 nm (line a), indicating the catalytic performance of CoFe-LDH in this system. In contrast, the TMB solution exhibits no color change in the absence of GOx (line d), illustrating that CoFe-LDH cannot oxidize TMB directly. The above results suggest that the hierarchical CoFe-LDH-NSA has a peroxidase-like catalytic ability, which catalyzes the oxidation of TMB from colorless to blue in the presence of glucose and GOx. Fig. 3B shows the UV-vis absorption spectra and corresponding photographs of the detecting solution with various concentrations of glucose (from 1 μM to 800 μM) after being catalysed by the Ni wire/CoFe-LDH-NSA. The intensity of the absorption peak at 652 nm increases gradually along with the enhancement of glucose concentration. As shown in the calibration curve (absorbance at 652 nm *versus* glucose concentration), a linear range is given from 1 μM to 20 μM with a detection limit of 0.47 μM (based on 3 SD/N) (Fig. 3C). To investigate the selectivity of this colorimetric glucose sensor, the catalysis performance of the CoFe-LDH-NSA toward DA, AA and UA was studied. Fig. 3D shows that there is no color change in the presence of these three compounds, implying high selectivity toward glucose detection. The UV-vis spectra of 3 repeated tests reveal good reusability, with the absorbance maintaining ∼95% of its initial response (Fig. S8†).

**Fig. 3:**
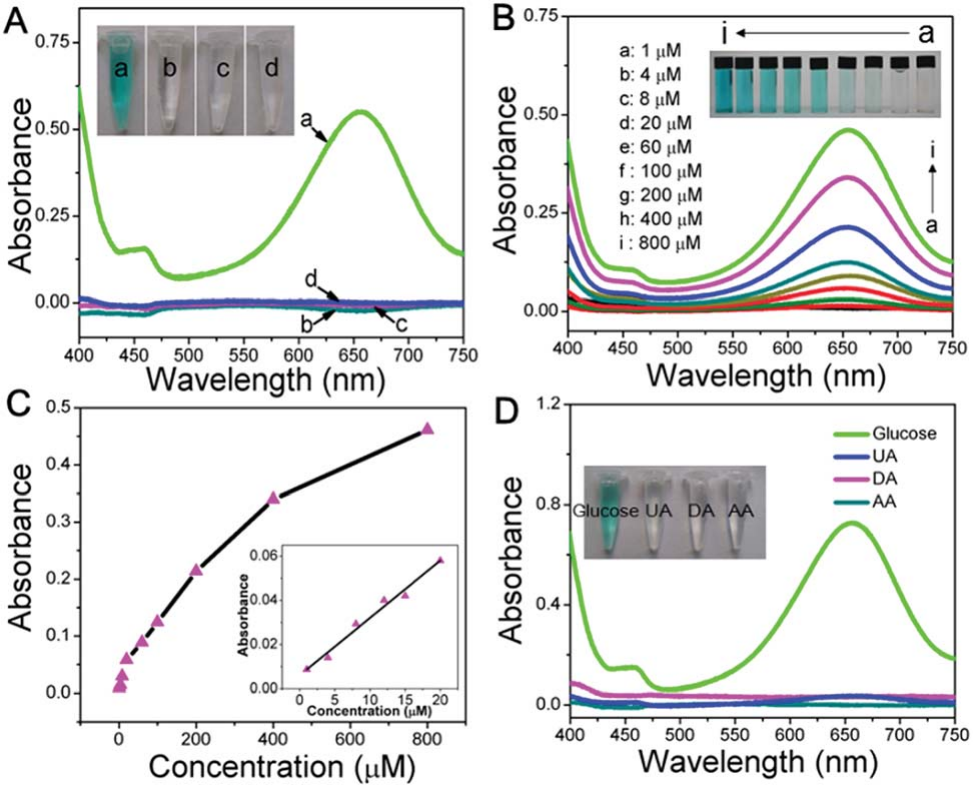
(A) UV-vis spectra of mixed solution containing: (a) TMB, GOx and glucose after reacting with the CoFe-LDH-NSA, (b) TMB, GOx and glucose without reacting with the CoFe-LDH-NSA, (c) TMB and glucose after reacting with the CoFe-LDH-NSA, (d) GOx and glucose after reacting with the CoFe-LDH-NSA. (B) UV-vis spectra and photographs for the CoFe-LDH colorimetric system with different glucose concentrations. (C) The calibration curve of absorbance at 652 nm *versus* glucose concentration from 1 μM to 800 μM. (D) Selectivity tests of the CoFe-LDH colorimetric system ([DA] = [AA] = [UA] = 5 mM, [glucose] = 2 mM).

Based on the glucose detection described above, the Ni wire/CoFe-LDH-NSA was applied to determine the concentration of glucose in human urine. As shown in Fig. 4A, the linear range for the electrochemical detection of glucose in diluted urine is nearly consistent with that measured in aqueous solution. To investigate the peroxidase-like colorimetric activity of the hierarchical LDH-NSA for urine glucose, UV-vis spectra of glucose (100 μM) in human urine (line c and d) and in aqueous solution (line a and b) are shown in Fig. 4B. The TMB solution exhibits the same color change in the presence or absence of urine in the glucose solution, with a linear range from 1 μM to 20 μM and a detection limit of 0.51 μM (based on 3SD/N), indicating good peroxidase-like colorimetric activity in human urine by using CoFe-LDH. Besides, it also possesses good protein anti-interference properties (Fig. S9†), indicating that the hierarchical CoFe-LDH-NSA is suitable for glucose analysis of real samples.

**Fig. 4:**
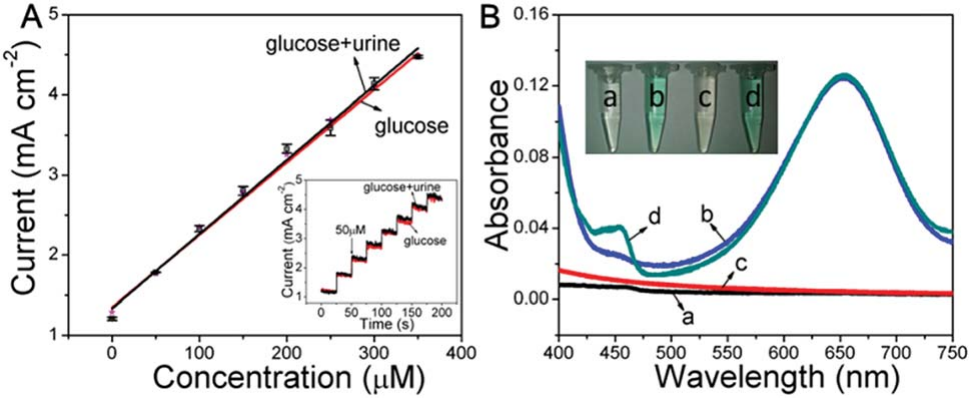
(A) Linear calibration plots for electrochemical glucose detection using CoFe-LDH in human urine and in aqueous solution, respectively (inset: the current–time curves measured at +0.4 V). (B) UV-vis spectra for colorimetric glucose detection in various solutions (a: glucose aqueous solution; b: with TMB; c: with human urine; d: with TMB and human urine).

## Conclusions

In summary, a bifunctional glucose sensor was fabricated by electrodeposition of the hierarchical CoFe-LDH nanosheet array on a Ni wire microsubstrate. The obtained Ni wire/CoFe-LDH-NSA exhibits remarkable activity in electrochemical and colorimetrical detection of glucose with a wide linear response range, high sensitivity and selectivity, and can serve as a multi-functional nonenzyme sensor with prospective applications.

## Experimental section

### Materials

Co(NO_3_)_2_·6H_2_O, FeSO_4_·7H_2_O, NaH_2_PO_4_·2H_2_O, Na_2_HPO_4_·12H_2_O, sodium acetate (NaAc), and KOH were purchased from Beijing Chemical Corp. Tetramethyl benzidine (TMB), glucose, dopamine (DA), uric acid (UA), ascorbic acid (AA), and alucose oxidase (GOx) were purchased from Aldrich Ltd. (Shanghai, China). The urine sample was obtained from a male volunteer without diabetes. All the chemicals were of analytical reagent grade and used without further purification. The water used was purified through a Millipore system.

### Preparation of 3D CoFe-LDH nanoarrays

The CoFe-LDH-NSA was synthesized on a Ni wire *via* a simple electrosynthesis method. Before synthesis, we first pretreated the Ni wire (150 μm in diameter) with HCl solution (2 M), absolute ethanol, and deionized water (each for 10 min) to remove the impurities or oxides on the surface. Then, the electrosynthesis process was operated in an electrochemical cell with a three-electrode configuration, including the working electrode (Ni wire), the counter electrode (Pt wire) and the reference electrode (saturated calomel electrode (SCE)). The electrolyte was a 50 mL aqueous solution containing Co(NO_3_)_2_·6H_2_O (0.15 M) and FeSO_4_·7H_2_O (0.15 M). The potentiostatic deposition was carried out at a potential of −1.0 V *vs.* SCE. The resulting Ni wire/CoFe-LDH-NSA was withdrawn and rinsed with distilled water. Co(OH)_2_ and FeOOH-NSAs were prepared *via* a similar electrosynthesis method by using different electrolytes: Co(OH)_2_ (Co(NO_3_)_2_·6H_2_O (0.15 M)); FeOOH (NaNO_3_ (0.15 M) + FeSO_4_·7H_2_O (0.15 M)).

### Characterization

A Shimadzu XRD-6000 diffractometer was used to collect the XRD patterns of the Ni wire/CoFe-LDH-NSA with a scan range between 3° and 70°. The SEM of Zeiss SUPRA 55 (accelerating voltage of = 20 kV) was used to investigated the morphology of the CoFe-LDH-NSA. The SEM was combined with EDS for the determination of metal composition.

### Glucose sensor measurements

#### Electrochemical sensor performance measurements

We used a CHI660E electrochemical workstation (Shanghai Chenhua Instrument Co., China) to carry out all the electrochemical experiments in this work. The measurements were operated in a three-electrode electrochemical cell at room temperature. The Ni wire/CoFe-LDH-NSA was directly used as the working electrode, and Pt wire and a SCE were used as the counter and reference electrode. The electrolyte was 0.1 M KOH aqueous solution with the addition of different concentrations of glucose.

#### Colorimetric sensor performance measurements

The colorimetric sensor performance of the Ni wire/CoFe-LDH-NSA for glucose was measured as per the following steps: (1) 200 μL of 2 mg mL^−1^ GOx and 200 μL of glucose with different concentrations were added in 400 μL of 20 mM Na_2_HPO_4_ buffer (pH = 7.0). Then the mixed solution was incubated at 37 °C for 2 h; (2) the Ni wire/CoFe-LDH-NSA (10 cm) was dropped into a 2 mL 0.5 mM NaAc buffer solution (pH = 4.0), followed by addition of 600 μL of TMB solution (1 mM in ethanol). Then 600 μL of the as prepared glucose-GOx solution were added into the above glucose reaction solution; (3) the resulting mixture was incubated for 30 °C for 30 min before the measurements. H_2_O_2_ detection was the same as the above glucose detection method, as long as the replacement of glucose-GOx solution with H_2_O_2_ of different concentrations. All the colorimetric experiments were carried out on a Shimadzu U-3000 spectrophotometer (the UV-vis absorption spectra were collected in the range 300−800 nm).

#### Detection of urine glucose

For the determination of glucose in human urine, firstly, we dissolved glucose (100 μM) in 20 mL of the urine sample obtained from a male volunteer without diabetes. Then, the as prepared sample was directly used for measurement.

## Statement of live subjects

Informed consent was obtained from all human subjects.

## Conflicts of interest

There are no conflicts to declare.

## Supplementary Material

NA-001-C8NA00231B-s001
