# Supplementary material for: A bifunctional nonenzymatic flexible glucose microsensor based on CoFe-Layered double hydroxide
Source: Nanoscale Adv. 2019 Jan 10;1(3):948–52. doi: 10.1039/c8na00231b (PMC9419660; doi:10.1039/c8na00231b)
Supplement: NA-001-C8NA00231B-s001 [file NA-001-C8NA00231B-s001.pdf]

Electronic Supplementary Material (ESI) for Nanoscale Advances.
This journal is © The Royal Society of Chemistry 2019

# Supporting Information for

## Bifunctional Nonenzymatic Flexible Glucose Microsensor based on CoFe-Layered Double Hydroxide

Junya Cui‡[1], Zhenhua Li‡[1], Ke Liu[1], Jianming Li[2], Mingfei Shao*[1]

[1]*State Key Laboratory of Chemical Resource Engineering, Beijing University of Chemical Technology, Beijing 100029, China*

[2]*Petroleum Geology Research and Laboratory Center, Research Institute of Petroleum Exploration & Development (RIPED), PetroChina, Beijing 100083, China*

‡ These authors contributed equally to this work.

* Corresponding authors. E-mail addresses: shaomf@mail.buct.edu.cn

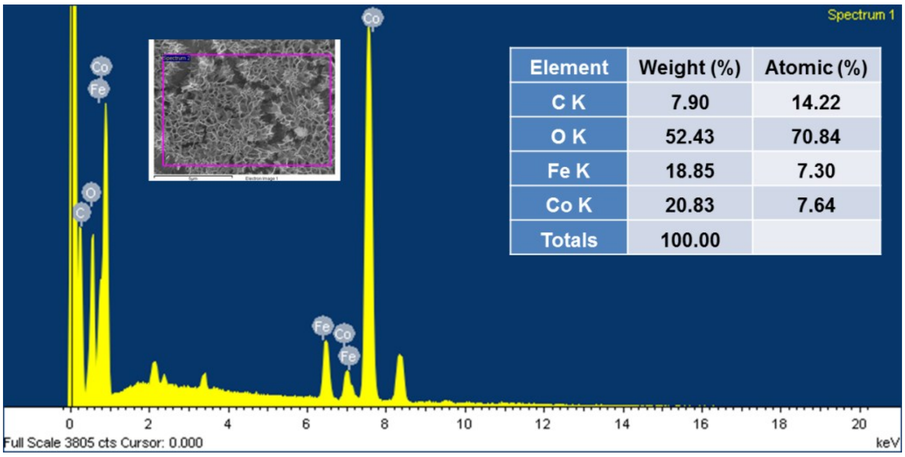


**Fig. S1** EDS spectra and corresponding elemental contents of Ni wire/CoFe-LDH-NSA.

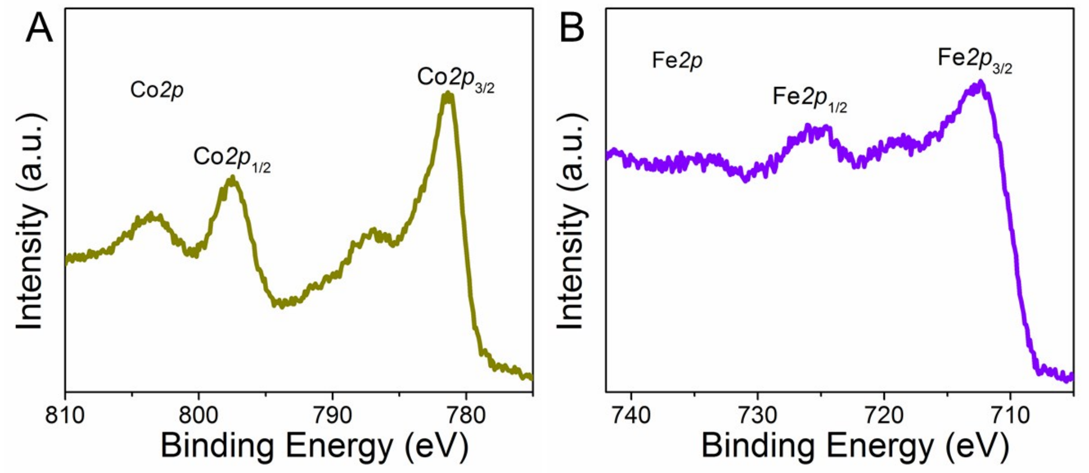


**Fig. S2** Typical XPS spectra of (A) Co 2*p* and (B) Fe 2*p* of Ni wire/CoFe-LDH-NSA.

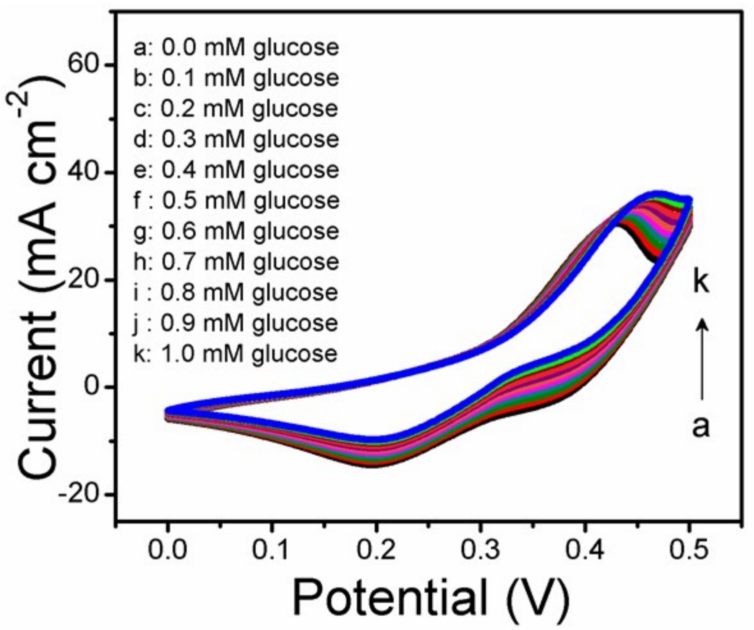


**Fig. S3** CVs of the Ni wire/CoFe-LDH-NSA in 0.1 M KOH solution at 10 mV $s^{-1}$ (the concentration of glucose from 0 mM to 1 mM).

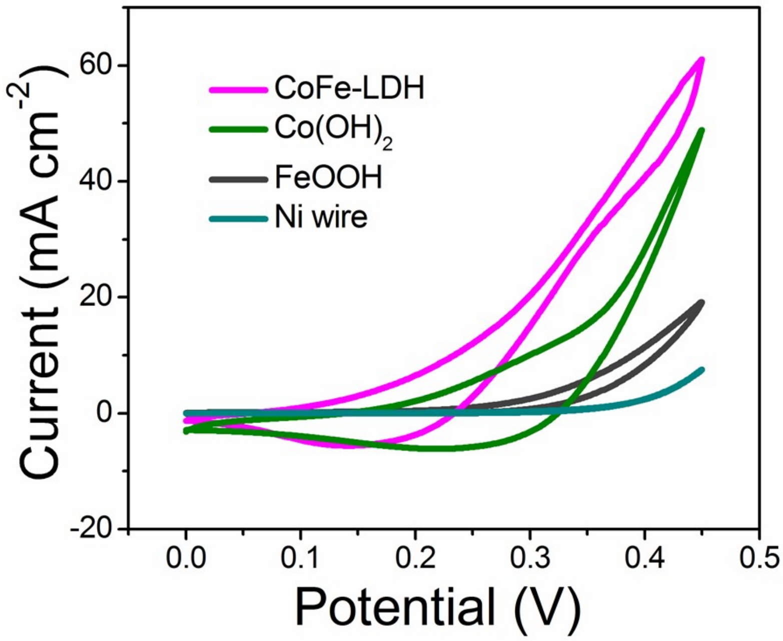


**Fig. S4** The CVs curve of Ni wire supported CoFe-LDH, $Co(OH)_2$, FeOOH, and pure Ni wire in 0.1 M KOH solution with 3 mM glucose at 10 mV $s^{-1}$.

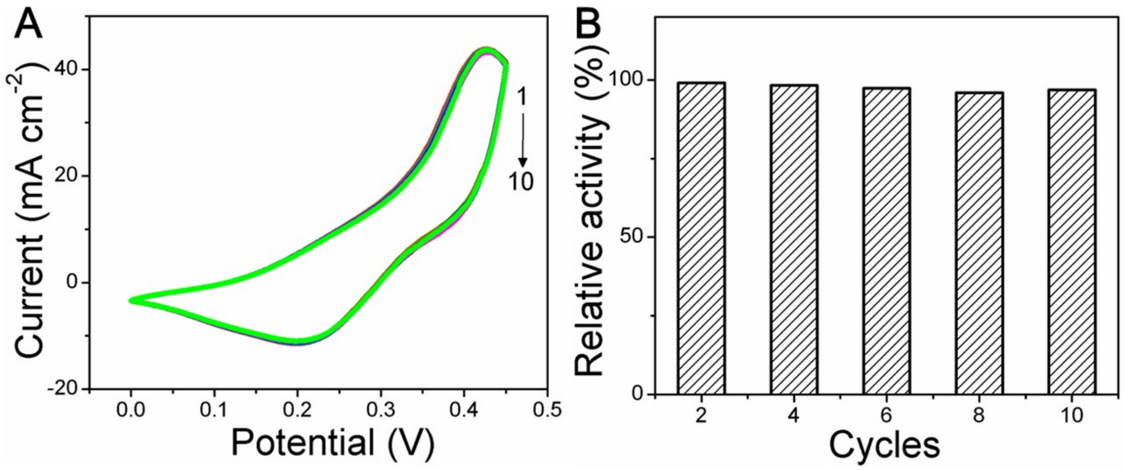


**Fig. S5** (A) The CVs curve of repeated cycle in 1.5 mM glucose solution (in 0.1 M KOH solution at 10 mV $s^{-1}$) and (B) the corresponding histogram.

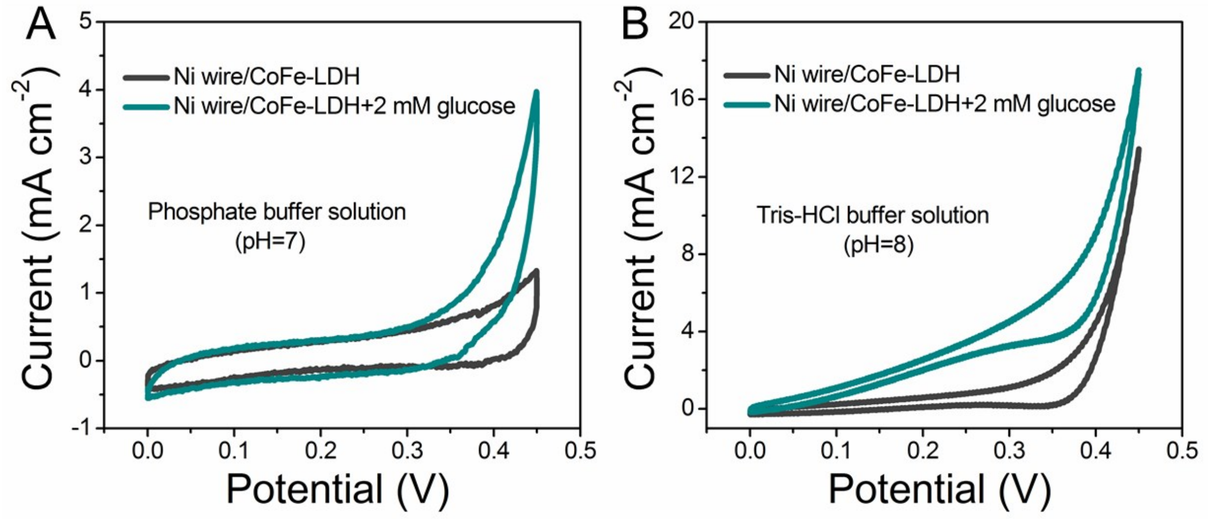


**Fig. S6** CVs of the Ni wire/CoFe-LDH-NSA in different buffer solution at 10 mV $s^{-1}$: (A) phosphate buffer solution, (B) Tris-HCl buffer solution.

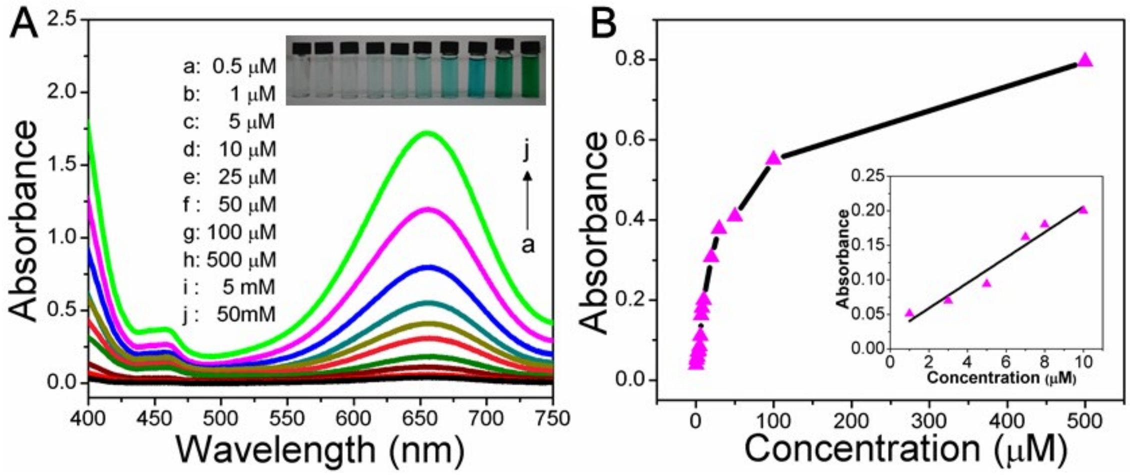


**Fig. S7** (A) UV-vis spectra and photographs for the CoFe-LDH colorimetric system with different $H_2O_2$ concentration (Incubation solution: 0.2 M NaAc buffer (pH=4.0) + 0.2 M TMB at 30 °C). (B) The calibration curve of absorbance at 652 nm versus the concentration of $H_2O_2$ from 0.5 μM to 500 μM.

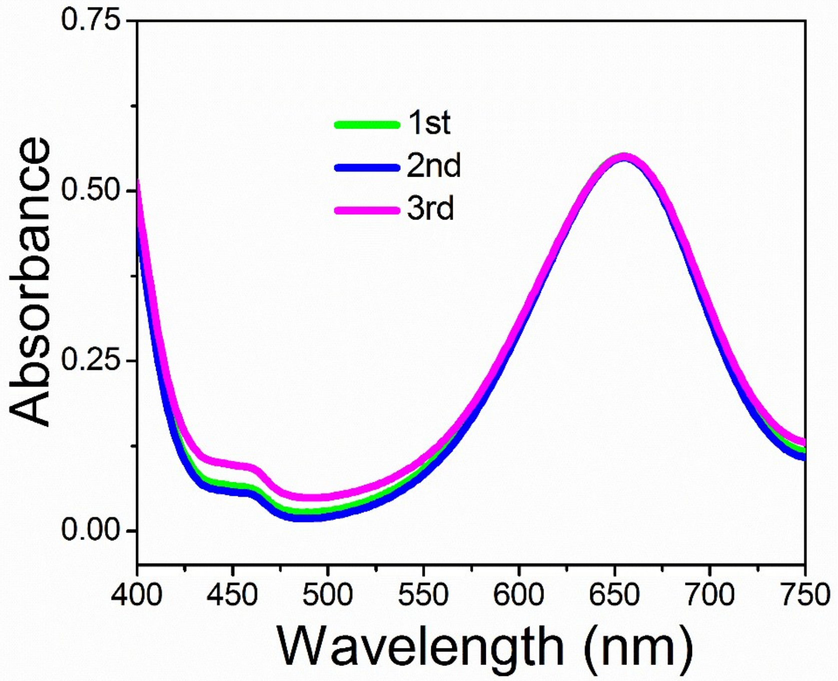


**Fig. S8** The UV-vis spectra of repeated cycle of 2 mM glucose solution.

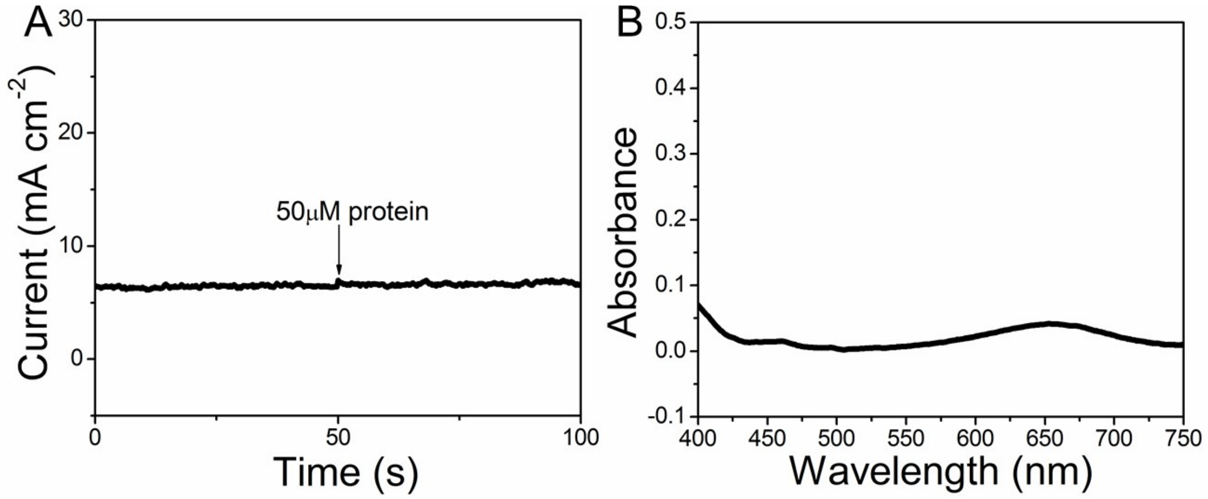


**Fig. S9** Protein anti-interference measurement of the Ni wire/CoFe-LDH-NSA: (A) electrochemical test in 0.1 M KOH at +0.45 V; (B) colorimetric test with 5 mM of protein. **There is no apparent response signal with the existence of protein in the sensing system, indicating good protein anti-interference property of the CoFe-LDH electrode.**

**Table S1.** Comparison of the performances of LDH-based non-enzymatic glucose sensors.

| Sensing material | Sensitivity ($mA\ mM^{-1}\ cm^{-2}$) | Limit of detection (μM) | Linear range (μM) | Refs. |
|---|---|---|---|---|
| **Ni wire/CoFe-LDH-NSA** | 1063 | 0.27 | 10−1000 | This work |
| **Ni Foam/NiFe-LDH** | 3680.2 | 0.59 | 2−800 | 1 |
| **CC/NiAl-LDH** | 14130 | 0.22 | 1−329 | 2 |
| **Ni Foam/NiCo-LDH** | 1235 | 1.6 | 1200−14800 | 3 |
| **Ti foil/NiAl-LDH** | 24.5 | 5 | / | 4 |
| **Au/NiAl-LDH/CNTs/Gr** | 1989 | 1 | 10−6100 | 5 |
| **NiAl-LDH/Nf/GCEs** | / | 0.11 | 1−100 | 6 |

## References


[1] Y. Lu, B. Jiang, L. Fang, S. Fan, F. Wu, B. Hu and F. Meng, *Electroanalysis*, 2017, **29**, 1.

[2] B. Hai and Y. Zou. *Sens. Actuat. B-Chem.*, 2015, **208**, 143.

[3] J. Chen, Q. Sheng, Y. Wang and J. Zheng. *Electroanal.*, 2015, **28**, 979.

[4] X. Li, J. Liu, X. Ji, J. Jiang, R. Ding, Y. Hu and X. Huang. *Sens. Actuat. B-Chem.*, 2010, **147**, 241.

[5] S. Fu, G. Fan, L. Yang and F. Li. *Electrochim. Acta*, 2015, **152**, 146.

[6] Z. Chen, J. Guo, T. Zhou, Y. Zhang and L. Chen, *Electrochim. Acta*, 2013, **109**, 532.
